# The Changing Prognosis of Ulcerative Colitis and Crohn Disease by Decades

**DOI:** 10.1093/crocol/otab008

**Published:** 2021-04-15

**Authors:** Burton I Korelitz, Judy Schneider

**Affiliations:** Inflammatory Bowel Disease Service, Division of Gastroenterology, Lenox Hill Hospital, New York, New York, USA

**Keywords:** ulcerative colitis, Crohn disease, decades, biologics, surgery, complications, cancer

## Abstract

We present a bird’s eye view of the prognosis for both ulcerative colitis and Crohn disease as contained in the database of an Inflammatory Bowel Disease gastroenterologist covering the period from 1950 until the present utilizing the variables of medical therapy, surgical intervention, complications, and deaths by decades.

## INTRODUCTION

The history and prognosis of any disease entity remain a work in motion, and this is clearly evident with the rapid increase in drugs available for the treatment of both ulcerative colitis (UC) and Crohn disease (CD). Accordingly, the period of time in which we have been involved in the study and management of inflammatory bowel disease (IBD) has seen outstanding changes. The span of observation concerned began in the 1950s and has extended to the present. To demonstrate the changes that have taken place we have arbitrarily chosen decades as the segments of time in which to document well-defined indicators of IBD activity and thereby prognosis.^1–5^

## METHODS

From our database of more than 3300 patients with IBD seen personally by the first author, we compared choice indicators of disease outcome from 6 decades: (1) 1961–1970, (2) 1971–1980, (3) 1981–1990, (4) 1991–2000, (5) 2001–2010, and (6) 2011 to the present, for both UC and CD. These indicators include (1) the number of IBD-related surgeries, (2) the number of related deaths, (3) the number of instances of toxic megacolon, considered a specific complication of both UC and CD, and (4) the occurrence of colon cancer, also considered specific for UC and CD. Note that the number of UC and CD patients was fairly stable across decades.

**FIGURE 1.**
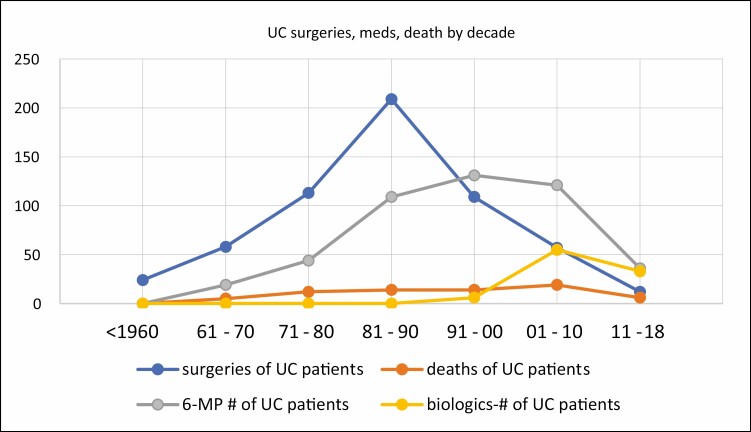
UC: drug treatment, surgeries, and deaths by decades.

**FIGURE 2.**
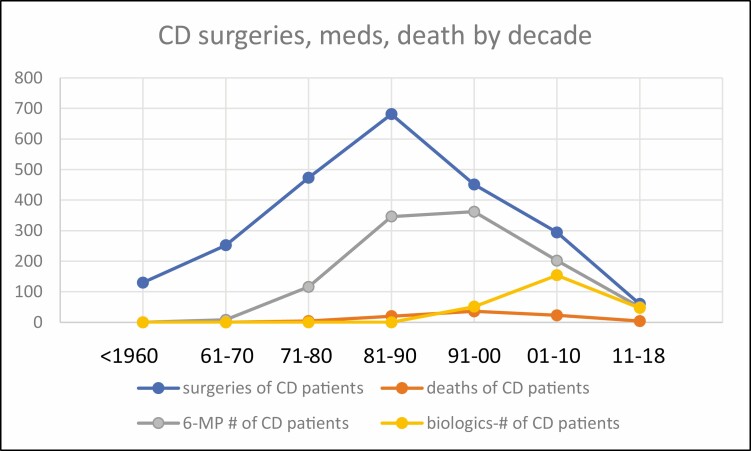
CD: drug treatment, surgeries, and deaths by decade.

**FIGURE 3.**
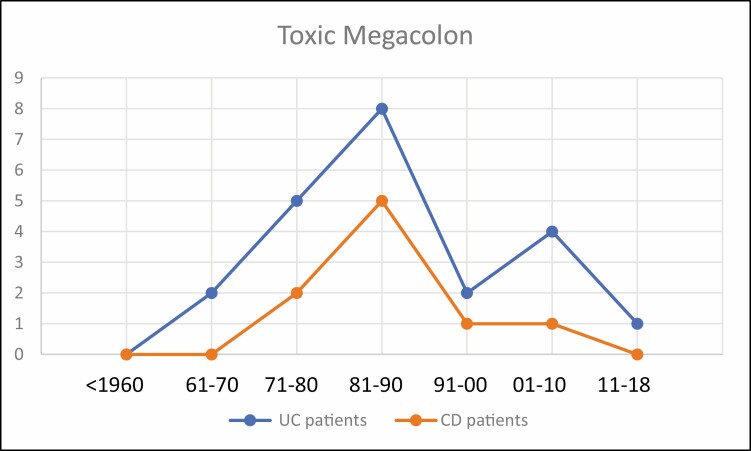
Toxic megacolon in UC and CD.

**FIGURE 4.**
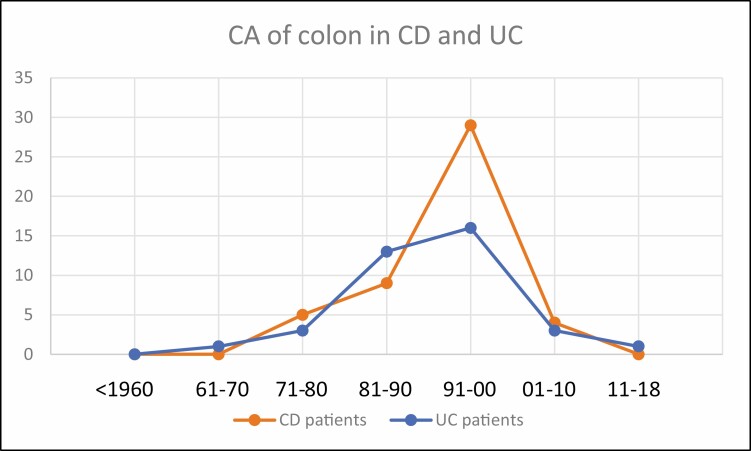
Cancer of the colon in UC and CD.

Table 1 shows the total numbers of patients in all 4 categories for each of the 6 decades for UC and Table 2 the same for CD. To both tables we have added data on treatment with the immunosuppressive drug 6-mercaptopurine (6-MP) which clearly had a favorable influence on the course of UC^6^ and CD,^7^ and also data on the treatment with biologicals, particularly infliximab, which had a favorable influence on both diseases starting in the late 1990s. These data are also present in graph form for each category in the tables (Figs. 1–4).

**TABLE 1.** Occurrence in the Course of UC by Decade

Decade	Surgeries	Deaths	Toxic Megacolon	Carcinoma of Colon	6-MP Rx	Biologics Rx
	No. Patients	No. Patients	No. Patients	No. Patients	No. Patients	No. Patients
<1960	24	0	0	0	0	0
1961–1970	58	5	2	1	19	0
1971–1980	113	12	5	3	44	0
1981–1990	209	14	8	13	109	0
1991–2000	109	14	2	16	131	6
2001–2010	57	19	4	3	121	55
2011 to present	12	6	1	1	36	33

**TABLE 2.** Occurrence in the Course of CD by Decade

Decade	Surgeries	Deaths	Toxic Megacolon	Carcinoma of Colon	6-MP Rx	Biologics Rx
	No. Patients	No. Patients	No. Patients	No. Patients	No. Patients	No. Patients
<1960	130	0	0	0	0	0
1961–1970	252	0	0	0	8	0
1971–1980	473	4	2	5	116	0
1981–1990	681	20	5	9	346	0
1991–2000	451	36	1	29	362	51
2001–2010	294	23	1	4	202	154
2011 to present	60	4	0	0	50	47

## RESULTS

The number of related surgeries for UC clearly reaches a peak in the decade 1981–1990 and fell thereafter. Patients died of complications of UC including surgical complications with a peak during the decade 2001–2010. The dramatic occurrence of toxic megacolon was most prevalent during 1981–1990, and the incidence of colon cancer, a late complication, during 1991–2000 (Figs. 3, 4). The use of 6-MP to treat UC also peaked from 1991 to 2010 and biologicals followed from 2001 to the present.

The number of CD-related surgeries peaked from 1981 to 1990 and then began to fall. Similarly, death from CD peaked from 1991 to 2000. Toxic megacolon complicating CD was never as prevalent as in UC, peaked in the decade 1981–1990, and carcinoma of the colon in CD was highest during 1991–2000 (Figs. 3, 4). The use of 6-MP was maximum from 1981 to 2000 whereas biologics dominated the field of therapy thereafter.

## DISCUSSION

During the time segment before 1960 drug management of both UC and CD was heavily weighted by treatment with sulfasalazine, 5 aminosalicylic acid products, adrenocorticotropic hormone, and corticosteroids, and supportive therapy with antibiotics and blood transfusions. During the next 3 decades, successful outcomes were heavily weighted by immunosuppressives, 6-MP more than azathioprine at our institution. Subsequently, the course of both UC and CD has been dramatically influenced by the treatment with biologicals, most often with infliximab, usually with continuation of the 6-MP.

Since the advent of 6-MP and subsequently biologicals, often used in combination, the number of hospitalizations (data not available) has dramatically fallen; in fact, the Fellows in Gastroenterology at our institution now see most of their IBD patients in the outpatient service. Similarly, the number of IBD-related surgeries has fallen markedly and emergency procedures have become rare. Patients rarely die any longer from UC or CD. Uveitis, which the senior author had often seen early in his IBD practice is now a rare phenomenon. This is also true of pyoderma gangrenosum and even erythema nodosum.

While the information presented here offers no surprises and all IBD clinicians have had similar experiences, we nevertheless document the changing and markedly improved prognosis for both UC and CD. Clearly, treatment with biologicals have contributed to a dramatically favorable outcome building on the earlier success of immunosuppressives, particularly 6-MP. Unfortunately, the causes of UC and CD remain unknown. When etiology catches up with successful disease management, perhaps IBD will become rare.

## Data Availability

Data are not publicly available.

## Notes

Funding: Funding provided by the New York Crohn’s Foundation.
